# Iron Deficiency and Iron Deficiency Anemia in Chronic Disease—Common, Important, and Treatable

**DOI:** 10.3390/jcm14134519

**Published:** 2025-06-26

**Authors:** Bjørn Moum, Stefan Lindgren

**Affiliations:** 1 Institute of Clinical Medicine, University of Oslo, 0316 Oslo, Norway; 2 Research Department, Østfold Hospital Trust Kalnes, 1714 Grålum, Norway; 3 Department of Clinical Sciences Malmø, Lund University, SE-221 00 Lund, Sweden; stefan.lindgren@med.lu.se

**Keywords:** iron deficiency, iron deficiency anemia, chronic disease, treatment

## Abstract

Iron has many important functions related to energy metabolism. However, hemoglobin synthesis is always a priority. Iron deficiency can be caused by increased loss, insufficient intake, or decreased absorption from the intestine and reduced release from depots in systemic inflammation. Anemia appears when stores are depleted or when utilization of iron from the stores is impaired. Treatment with oral iron is the first choice when the intestine is healthy, and the patient is free of inflammation. Intravenous iron is indicated when oral iron is ineffective or not tolerated and if more rapid correction is clinically indicated as in severe anemia not requiring transfusion.

## 1. Iron Deficiency and Anemia as a Global Health Problem

Iron deficiency is a significant global health problem that affects millions of people worldwide. It is particularly prevalent in low- and middle-income countries but can also affect the population of high-income countries.

The most common cause of anemia globally in 2021 was iron deficiency anemia, with a prevalence of 422 life-years per 100,000 people [1].

Iron deficiency can have serious consequences for health. When the body does not receive sufficient iron, hemoglobin synthesis is impaired, and anemia can lead to fatigue, weakness, and a reduced ability to perform daily tasks. Iron also has many other functions in the body, and iron deficiency has consequences in addition to those directly related to anemia.

One of the major challenges associated with iron deficiency is access to nutritious food, especially for vulnerable groups such as pregnant women, children, and people in low-income areas. Economic constraints, inadequate access to a varied diet, especially decreasing meat consumption, and cultural factors can all contribute to the prevalence of iron deficiency [2,3].

Prevention and treatment of iron deficiency are important to improve global health. This may include measures for vulnerable groups such as the elderly, dietary adjustments, fortification of foods with iron, and in some cases, iron supplementation. Of particular importance is gastrointestinal iron loss due to colorectal cancer in the elderly. In addition, it is important to address the social causes of iron deficiency, such as access to health services, education about nutrition, and economic conditions and to avoid the negative effects on iron absorption from the overuse of proton pump inhibitors [4,5].

Even in high-income countries, with developed health services and varied dietary habits, there are groups of the population that are at risk of iron deficiency. Menstrual blood loss is the most important cause of iron deficiency in premenstrual women [6]. It is therefore important to be aware of the problem and to diagnose and implement measures to prevent and treat it.

In many chronic inflammatory diseases, iron deficiency has consequences for the health of patients and the course of the disease. Intravenous iron therapy has been shown to have positive effects on the quality of life for such patients and for the course of the disease [7].

## 2. The Importance of Iron and Iron Metabolism

Iron is the most important trace element in the organism [8]. In its inorganic form, iron can exchange between Fe2+ and Fe3+, which gives iron unique properties for important redox reactions such as binding oxygen in hemoglobin, activating metabolic enzyme systems, and driving energy production. Free Fe2+ can induce the formation of free radicals that lead to damage to lipid membranes, proteins, and DNA. Therefore, free Fe2+ must be kept low. Several different forms of iron-binding proteins therefore effectively regulate iron metabolism.

**Figure 1:**
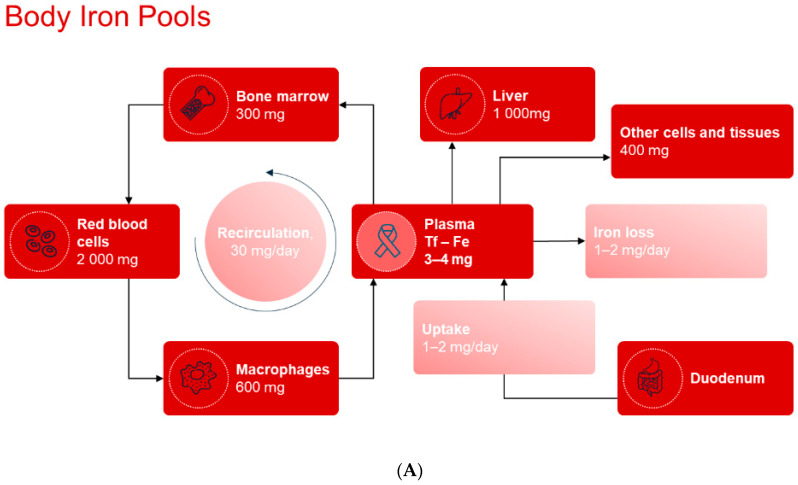

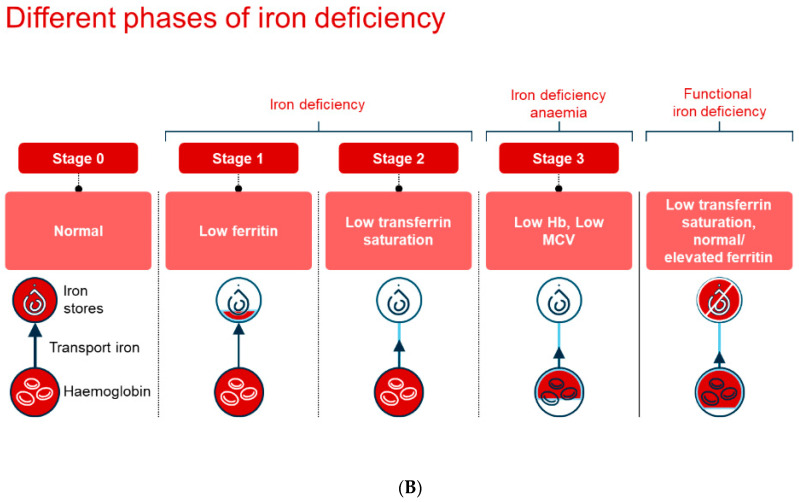
(**A**) Iron distribution and turnover. (**B**) Different stages of iron deficiency.

The normal daily requirement for iron is 1–2 mg. The body’s iron is distributed as 60% bound in hemoglobin, 25% stored in ferritin, while 15% is bound in myoglobin, enzymes, and transferrin (Figure 1A). The elimination of iron occurs primarily through the breakdown of intestinal epithelial cells where iron is bound to ferritin. Losses increase with systemic inflammation when a larger amount of iron is stored in enterocytes. Iron is recycled after the destruction of erythrocytes in the body’s macrophages, and iron is transported bound to transferrin for functions in various organ systems. The absorption of iron is controlled by the peptide hepcidin, which regulates access to iron during erythropoiesis.

## 3. Anemia of Chronic Disease

Anemia of chronic disease, also known as anemia of inflammation, occurs as a direct result of immune activation in the body. This immune activation can be triggered by several factors, including infections, long-term inflammatory diseases, malignancies that do not result in bone marrow infiltration, and chronic renal failure, particularly in patients undergoing dialysis. Although anemia of chronic disease is a well-known medical condition, there are few reliable figures that accurately indicate its prevalence. The prevalence varies considerably between different studies, ranging from as low as 8% to as high as 95% [9,10].

It is also important to note that the incidence of anemia tends to increase with age. Among older adults, anemia of inflammation is the most common form of anemia detected. An American study found that approximately 10% of the population over the age of 65 suffers from anemia. When looking at this group of elderly people, it turned out that 17% of them had iron deficiency anemia, while 20% suffered from anemia related to chronic disease. Only a small proportion, about 6%, had anemia caused solely by vitamin B12 deficiency. In addition, an explanation for the anemia was not found in as many as 34% of the patients in this study [11].

Anemia usually develops gradually over a longer period and can often be the first sign of an underlying disease (Figure 1B). Usually, the symptoms of the underlying disease dominate the patient’s medical history, which means that anemia is often discovered as an incidental finding during medical examinations. Common causes of anemia include long-term inflammation or acute infections, cancer, kidney disease, and liver disease. The mechanisms underlying anemia in chronic diseases are many and often occur in parallel. The most common causes are poor nutrition and deficiencies of iron, vitamin B12, and folic acid.

Furthermore, anemia is seen in the context of renal failure due to reduced erythropoiesis, a consequence of reduced erythropoietin stimulation. In heart failure, anemia can occur due to the reduced intake and reduced absorption of nutrients due to low-grade inflammation and an edematous bowel, combined with increased iron loss often related to the use of platelet inhibitors and anticoagulants. The treatment of acute severe anemia through a blood transfusion may, in some cases, be necessary to raise hemoglobin levels when these reach dangerously low levels.

In cases where anemia is caused by inadequate nutrition, it is of crucial importance that the affected person has access to nutritious food containing enough iron and essential vitamins. Iron treatment should only be initiated when iron deficiency is confirmed, since iron supplements in the absence of iron deficiency can worsen existing inflammatory conditions. For patients with certain diseases, supplementary treatment with iron, erythropoietin, or vitamins may be necessary. In addition, the anemia is likely to improve when the underlying disease is treated.

## 4. Iron Deficiency Anemia in Chronic Disease

**Figure 2:**
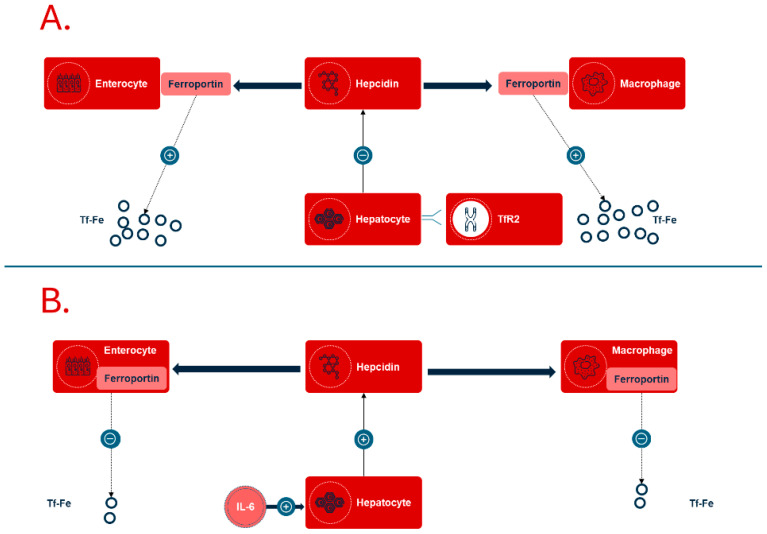
The role of the peptide hepcidin in iron metabolism. (**A**) Several receptors on the hepatocyte surface, among which is the transferrin receptor 2 (TfR2), react to the circulating amount of transferrin-bound iron. In iron deficiency, the production of hepcidin is downregulated, and the absorption of iron from the intestine and release from stores, mediated by ferroportin, increases. (**B**) In systemic inflammation, represented by IL-6, the production of hepcidin increases. Ferroportin is internalized and inactivated, and the availability of iron from the intestine and stores is strongly reduced, resulting in functional iron deficiency.

Iron deficiency anemia associated with chronic diseases is a condition in which the body does not have enough iron, or the available iron is unable to support erythropoiesis [12]. This leads to functional iron deficiency, where an increased production of hepcidin in the liver is stimulated by chronic inflammatory processes (Figure 2).

This relationship has been linked to the production of proinflammatory cytokines, especially interleukin-6 (IL-6), which is known for its role in inflammation. Increased hepcidin levels inhibit both the absorption of iron in the intestine and the release of iron from macrophages. This inhibition occurs since hepcidin affects the degradation of the iron export protein ferroportin, which is found in the cell membranes of enterocytes and macrophages. The result of this mechanism can be a significant reduction in iron availability, even when there is sufficient iron present in the diet and in body stores. The primary cause of the development of anemia in chronic conditions, such as chronic kidney disease or rheumatoid arthritis, is therefore an excessive activation of the inflammatory response [13,14].

Furthermore, intestinal bleeding can also be a contributor to iron deficiency. This bleeding can be a result of damage to the intestinal mucosa because of chronic diseases. It is also worth mentioning that iron deficiency is particularly prevalent among obese patients. This is because obese individuals often have increased circulating levels of acute-phase reactants because of adiposity-associated inflammation. This condition leads to reduced iron absorption that correlates with an inhibition of duodenal ferroportin expression, while at the same time, hepcidin concentrations increase. Therefore, inflammation in obese individuals is closely linked to the occurrence of iron deficiency anemia. It has been observed that people who undergo bariatric surgery often experience a reduction in the inflammatory response, which is accompanied by lower levels of hepcidin, which in turn results in improved iron absorption. On the other hand, surgical procedures such as gastric bypass and sleeve gastrectomy can cause malabsorption in the small intestine, which can lead to iron deficiency in these patients [15].

In the treatment of iron deficiency anemia associated with chronic diseases, great emphasis is often placed on addressing the underlying cause of the iron deficiency. This usually involves treating the chronic disease responsible for the problem. Iron supplementation is often considered necessary to compensate for the iron deficiency, but there is a major challenge in adapting the treatment to the underlying disease. Because inflammation via hepcidin blocks iron absorption from the intestine, as well as the release from iron stores, intravenous treatment should be considered the preferred option in such cases.

It is also important for patients to see a healthcare professional, including specialists such as gastroenterologists or hematologists, to obtain an accurate diagnosis and a tailored treatment plan. Such a plan should be based on individual needs and symptoms, making it crucial to ensure that the patient receives the most appropriate treatment for their specific situation.

## 5. Iron Deficiency Anemia and Fatigue

The relationship between iron deficiency and chronic fatigue and the significance of this condition has been the subject of extensive debate amongst professionals. Despite this, there is no doubt about the negative effects that iron deficiency and anemia can have on an individual’s life, which include chronic fatigue, cognitive impairment, reduced physical function, impaired development of various physical and mental capacities, and a generally poor health-related quality of life [16]. These consequences can affect both daily life and opportunities to participate in work and social activities.

Iron deficiency without anemia is a widespread condition, where the prevalence among premenopausal women is estimated to vary between 16% and 23%. In regions such as the Middle East, the figure can be as high as 58% in women aged 18 to 50 years old who suffer from isolated iron deficiency without necessarily having developed anemia [17]. This shows that iron deficiency is a global challenge that can affect many people, regardless of the level of development of health services.

Research has shown that iron deficiency without anemia can lead to significant health problems such as fatigue, cognitive impairment, and reduced physical endurance [18,19,20]. Treatment in the form of iron supplements or dietary changes can help improve these conditions. However, the gradual transition from iron deficiency to iron deficiency anemia presents a significant challenge, as the symptoms are often non-specific. This makes it difficult to determine the extent of the problem with isolated iron deficiency, and therefore the criteria for treatment are also far from being clarified. It is crucial, as mentioned earlier, to identify the cause of the iron deficiency before implementing treatment methods. This is necessary to uncover and address any underlying causes that may lead to a relapse of the condition.

At the same time, treatment should be tailored to individual needs and tolerance levels to avoid potential side effects that may arise from the use of supplements. It is important to note that people with iron deficiency without anemia who should be considered for treatment include those who have low ferritin levels or low transferrin saturation (TSAT), which is a known indicator of the availability of iron in the body. This may also include people who show symptoms of iron deficiency, even if they do not meet the specific criteria for what defines anemia.

Pregnant women are a special risk group and are often treated for iron deficiency even before anemia develops, as iron is essential for fetal development. It is important to point out that the need for iron during this period is at least twice as high as it was before pregnancy. Several international guidelines stress the importance of detecting iron deficiency without waiting for anemia to occur [21]. Treatment may involve the use of iron supplements, as well as changes in the diet to include foods with higher iron content. Intravenous iron treatment is rarely necessary, which means that in most cases the treatment can be managed in a more gentle and effective way [22]. In addition, people with chronic diseases such as celiac disease, inflammatory bowel disease, heart failure, or kidney disease can also significantly benefit from iron treatment, even if they have not developed anemia.

By understanding the complexities surrounding iron deficiency and its impact on health, we can better implement the necessary measures and treatments for affected individuals, thereby improving their quality of life.

## 6. Diagnosis of Iron Deficiency and Iron Deficiency Anemia

Diagnosis of iron deficiency and iron deficiency anemia can be a significant challenge, and it is important to note that the biochemical tests commonly used are not always able to unambiguously distinguish between iron deficiency anemia and anemia caused by chronic disease (see Figure 1B for illustration). In a healthy population, ferritin levels are usually a sensitive and reliable marker for assessing iron status, but in the presence of inflammation, ferritin acts as an acute-phase reactant and serum levels increase. This adds to the complexity of the diagnosis, since even normal or moderately increased levels in ferritin may still be compatible with iron deficiency.

Furthermore, dietary iron absorption is also affected by proinflammatory cytokines that lead to an increased production in hepcidin. Hepcidin is a peptide that regulates iron metabolism by limiting the absorption of iron in the intestine, and this may mean that patients with chronic inflammation may have limited iron availability. This can occur despite showing normal or even high levels of ferritin; therefore, the usual threshold for iron deficiency, defined as a ferritin level below 30 μg/L (see Table 1), does not apply. In such cases, transferrin saturation percentage (TSAT) levels should also be included in the assessment. It is obvious that ferritin levels between 100 μg/L and 300 μg/L require a TSAT value below 20% to confirm the presence of iron deficiency.

Routine monitoring of both ferritin and TSAT in groups at risk of iron deficiency is strongly recommended, as this may help to detect disease states at an early stage. It should also be underlined that the measurement of plasma iron alone has very limited value in the diagnostic process. In addition, the soluble transferrin receptor (sTfR) is a useful tool in this context, as it is not affected by either chronic or acute inflammation. This gives sTfR a high degree of sensitivity and specificity when diagnosing iron deficiency. Since sTfR is influenced by erythropoietic activity, calculation of the sTfR/log ferritin index is necessary for optimal utility, adding complexity to interpretation. In this context, Reticulocyte Hemoglobin Equivalent (Ret-He) is emerging as a promising and underutilized biomarker [23]. Ret-He directly reflects the hemoglobin content in reticulocytes, thereby providing a near real-time assessment of functional iron availability for erythropoiesis. Importantly, it remains largely unaffected by inflammation, making it particularly valuable in chronic disease settings when hepcidin-driven iron sequestration skews conventional markers. Despite high sensitivity and specificity for iron-deficient erythropoiesis, Ret-He is still not universally adopted in clinical algorithms, possibly due to a limited familiarity among clinicians. It is important to keep in mind that even if the hemoglobin (Hb) level is normal, iron deficiency cannot be ruled out, as anemia is a late complication of the underlying iron deficiency condition [24]. Therefore, it is crucial to have a thorough approach to the diagnosis and monitoring of the iron status, especially in patients with risk factors for iron deficiency and inflammation. We suggest that Ret-He should be integrated into the standard diagnostic work-up for patients with suspected iron deficiency in the setting of chronic disease.

## 7. Treatment of Iron Deficiency and Iron Deficiency Anemia

Oral iron is a safe, affordable, and simple method of administration for healthy individuals. But there are also significant challenges associated with oral iron therapy, since many patients experience side effects. Furthermore, treatment often needs to be continued for up to six months to normalize hemoglobin (Hb) levels and adequately replenish body iron stores. In addition, oral iron supplementation may contribute to increased intestinal inflammation in individuals with chronic inflammatory bowel disease, potentially leading to exacerbation of the disease [25]. It is important to keep in mind that systemic inflammation, such as in active disease where an increase in C-reactive protein (CRP) is observed, may lead to further hepcidin blockades that prevent effective oral iron absorption.

An interesting new development in oral iron therapy is ferric maltol (Ferracru^®^), which is a new, non-salt oral iron formulation. This formulation is specially designed with stable iron (III) complexed with a sugar derivative, tri-maltol. The unique sugar binding of iron reduces the formation of free iron in the body, which in turn increases the bioavailability and thus the effectiveness of iron treatment. Furthermore, it has also been suggested that ferric maltol has a less negative effect on the gut microbiome, which may be an advantage. However, it is important to note that according to the joint catalog text, this preparation is not recommended for patients with chronic inflammatory bowel disease during active flare-ups, or in patients who have hemoglobin values below 9.5 g/dL.

Intravenous iron therapy offers a quick and safe solution to normalize hemoglobin levels and restore iron stores in the body. In contrast to oral preparations, intravenous iron works independently of any concomitant systemic inflammation, making it an attractive alternative in many clinical situations. In Europe and the United States, ferric carboxymaltose and ferric derisomaltose are mainly used, both of which have been shown to be equally effective in the treatment of iron deficiency. Other preparations, such as iron sucrose and low-molecular-weight iron dextran, are also available and still widely used in the US but infrequently in Europe.

To calculate the total iron requirement, clinicians consider factors such as ferritin values, hemoglobin, and the patient’s body weight. There is almost no reason to give doses of less than 1 g to adult patients, as the risk of overtreatment is extremely low, while cases of undertreatment often occur. Six to eight weeks after the total dose has been administered, the efficacy should be assessed by measuring ferritin, transferrin saturation, and hemoglobin. Thereafter, it is important to monitor the patient’s hemoglobin level and iron stores regularly, in accordance with the underlying disease, and to re-treat before iron deficiency or anemia recurs. All preparations used are generally safe, and the frequency of serious allergic reactions is very low, with an incidence of approximately 1 in 250,000 doses administered [26].

Nevertheless, approximately 1% of patients experience some type of adverse reaction after each dose. Most of these are non-allergic in nature, related to complement activation on free iron particles (Complement Activation Related Pseudo Allergy, CARPA, or Fishbane reaction), and usually occur immediately after the infusion has been started. In most cases, these adverse reactions are rapidly transient, especially when treatment is stopped. The infusion can then be resumed at a lower infusion rate to minimize the risk of recurrence of the adverse reactions [27]. It is essential that both patients and healthcare professionals are well informed about the common adverse reactions and their generally harmless nature. It is also essential to follow the instructions regarding the dilution of the iron solutions, as dilution in more saline than recommended may lead to an increased amount of free iron and thus a higher risk of adverse reactions. In addition, the risk of hypophosphatemia with the lowest concentrations after two weeks should be observed, particularly when repeated doses of ferric carboxymaltose are administered [28].

## 8. Conclusions

- Iron is crucial for energy metabolism, and deficiency can negatively impact health-related quality of life. Hemoglobin synthesis takes priority over other bodily processes when iron is scarce.
- Iron deficiency arises from increased iron loss, insufficient dietary intake, impaired intestinal absorption, or hepcidin blockade (hindering both iron absorption and release from stores during systemic inflammation) and appears when stores are depleted or when mobilization of iron from stores is impaired. These distinct causes necessitate tailored diagnostic and therapeutic strategies.
- Anemia is a late-stage manifestation of iron deficiency, typically appearing only after iron stores are exhausted. Diagnosis of iron deficiency anemia relies on hemoglobin (Hb), ferritin, and transferrin saturation (TSAT) measurements. Soluble transferrin receptor (sTfR) and Reticulocyte Hemoglobin (Ret-He) assessment can be helpful, especially in inflammation-related cases where ferritin levels may be misleading, potentially masking severe iron deficiency.
- Oral iron therapy is generally the first-line treatment for patients with normal gut function and without underlying inflammation. However, side effects can limit adherence, and treatment often requires up to six months to normalize hemoglobin and replenish iron stores. If oral iron is ineffective, particularly in patients with systemic inflammation, or is poorly tolerated, intravenous iron offers a rapid, effective, and safe alternative for immediate iron repletion.
- Intravenous iron provides effective, rapid, and safe treatment when oral treatment is ineffective or not tolerated and if more rapid correction is clinically indicated as in severe anemia not requiring transfusion.

### Further Directions

More detailed guidelines are needed for diagnosing absolute iron deficiency in chronic inflammatory conditions. Ret-He is a promising tool that could further support indications for and dosing of intravenous iron in these patients. Additional clinical data are needed regarding outcomes such as the effects of iron deficiency treatment on the disease process, quality of life, sick leave, hospitalization, and cognitive capacity.

## Figures and Tables

**Table 1:** **Diagnostic criteria for iron deficiency anemia.**

Diagnostic for lower normal levels in iron deficiency anemia	
Hemoglobin	<13.0 g/dL for men
	<12.0 g/dL for women
	<11.0 g/dL for pregnant women
Ferritin *	<30 µg/L without inflammation (CRP ** normal)
	<100 µg/L with inflammation (CRP ** elevated)
Transferrin ***	Increased (or normal to low in inflammation)
Total iron-binding capacity	Increased (or normal to low in inflammation)
Transferrin saturation (TSAT)	<20%
MCV	Low (or normal in inflammation)
sTfR ****	High

It is important to emphasize that the hemoglobin levels indicated are lower limits according to the WHO, and that healthy men and women are usually in the range of 13–15 g/dL. * Acute-phase protein that may be increased in inflammatory conditions. ** CRP: C-reactive protein. *** Acute-phase protein that may be normal or increased in inflammation. **** sTfR: Soluble transferrin receptor.
